# Quality of life in pregnancy after percutaneous closure of atrial septal defect guided by transthoracic echocardiography

**DOI:** 10.1186/s12872-022-02995-x

**Published:** 2022-12-17

**Authors:** Zhi-Huang Qiu, Zhen-Fang Zong, Qing-Song Wu, Jun Xiao, Tian-Ci Chai, Xiao-Dong Chen, Yue Shen, Liang-Wan Chen

**Affiliations:** 1 Department of Cardiovascular Surgery, Union Hospital, Fujian Medical University, Fuzhou, 350001 Fujian China; 2 Key Laboratory of Cardio-Thoracic Surgery, Fujian Medical University, Fujian Province University, Fuzhou, China; 3 Fujian Provincial Special Reserve Talents Laboratory, Fuzhou, China; 4 Department of Cardiology, Anyang Regional Hosptial, Puyang, Henan China

**Keywords:** Quality of life, Pregnancy, Atrial septal defect, Transthoracic echocardiography guided percutaneous closure

## Abstract

**Purpose:**

We evaluated quality of life (QoL) in pregnant women who underwent transthoracic echocardiography-guided percutaneous closure of atrial septal defect (ASD).

**Methods:**

A total of 45 pregnant women underwent transthoracic echocardiography-guided percutaneous closure of ASD. We assessed QoL using the 36-Item Short Form Survey (SF-36) and compared results between pre- and post-procedure patients, as well as between those with ASD and healthy women in their second and third trimesters of pregnancy.

**Results:**

All patients showed improved right ventricular function and were classified as Class I, post-procedure. Mean SF-36 scores of the post-procedure group were better on all sub-scales than those of the pre-procedure group (*p* < 0.05), with the exception of role-emotional and mental health. Mean SF-36 scores for the pre-procedure group were also lower on all sub-scales than those of healthy pregnant controls (*p* < 0.05), with the exception of role physical, role emotional, and mental health. There was no difference between the post-procedure group and healthy pregnant controls. In a subgroup analysis, scores were better in some dimensions (social functioning and role emotional) for post-procedure patients in the 31–40 years of age group and the group on their second or third pregnancies than those of the 20–30 years of age group and the group on their first pregnancies (*p* < 0.05).

**Conclusion:**

After closure of ASD, QoL in pregnant women was improved. In a subgroup analysis, the younger women and those on their first pregnancy performed more poorly in some dimensions (social functioning and role emotional); this suggested that these groups should receive more proactive intervention.

## Plain English summary

Limited data was available on the general quality of life (QoL) in pregnant women with atrial septal defect (ASD), even though the condition could produce anxiety over health of the pregnancy and fetus. The percutaneous closure procedure was available for ASD during pregnancy; however, pregnant women were often concerned that the required X-rays would harm the fetus. A safe and effective procedure, percutaneous closure of ASD guided by transthoracic echocardiography, was widely used for this condition. This study used the 36-Item Short Form Survey (SF-36) to assess QoL in pregnant women with ASD pre- and post-procedure and compared the results to those of healthy pregnant women at a similar stage of pregnancy. Post-procedure QoL in pregnant women with ASD was improved; however, the younger women and those on their first pregnancy performed more poorly in some dimensions (social functioning and role emotional). Our results suggested that these groups should receive more proactive intervention.

## Introduction

Atrial septal defect (ASD) is a common congenital heart disease with a morbidity rate of 1/1000 [1]. Patients with ASD could go misdiagnosed if they did not present with clinical symptoms. However, when women entered pregnancy, circulating blood volume might increase by 40%–45%. At 32–34 weeks gestation, blood volume and cardiac load might reach a peak [2, 3]. In pregnant women with ASD, the left-to-right shunt was increased and the right atrium and right ventricle enlarged, which might lead to pulmonary hypertension and heart failure. Thus, for mis- or undiagnosed female patients of childbearing age, an ASD diagnosis often took place during pregnancy due to decreased cardiac function.

With the development of interventional technology, percutaneous closure of ASD guided by transthoracic echocardiography was now widely used in clinical practice. At present, many studies had evaluated the safety and efficiency of the technique [4–6] but scarce data had been reported about the quality of life (QoL) in pregnant women with ASD before and after this procedure. Depression and fear of disease might have a negative impact on the pregnant women’s QoL, and the curing of the disease might increase confidence and QoL.

In this study, we used the 36-Item Short Form Survey (SF-36) to assess QoL in pregnant women with ASD before and after procedure and compared the results with those of healthy women at a similar stage of pregnancy.

## Methods

### Patients

From January 2017 to January 2022, 45 pregnant women with ASD underwent percutaneous closure of ASD guided by transthoracic echocardiography. Right ventricular function was evaluated by echocardiography before and after the procedure, and 45 healthy pregnant women at similar stages of pregnancy were used as controls. Baseline characteristics were shown in Table 1. All pregnant women were diagnosed with ASD during the second trimester (20–24 weeks); the procedures were also performed during this period. The procedure indications for pregnant women with ASD were: (1) dyspnea symptoms; (2) cardiac function was grade II-III; 3) echocardiography indicated that the right ventricular function was impaired.

**Table 1:** Demographic characteristics of patients with atrial septal defect in pregnancy and healthy pregnant controls

Characteristics	Pateints (*n* = 45)	Controls (*n* = 45)	*P* value
Age(years)			
20–30	26	24	0.67
31–40	19	21	0.67
Marital status			
Married	45	45	N/A
Unmarried	0	0	N/A
Number of pregnancy			
First	23	27	0.40
Second or third	22	18	0.40
NYHA			
I	10	43	< 0.001
II	33	2	< 0.001
III	2	0	N/A
IV	0	0	N/A

*NYHA* New York Heart Association (classification)

### SF-36 Health Survey

The SF-36, used to assess the health-related QoL, consists of eight domains: physical functioning, role physical, bodily pain, general health, vitality, social functioning (SF), role emotional (RE), mental health [7].

The survey was conducted in the outpatient department of cardiovascular surgery for the patient group and in the outpatient department of obstetrics for the control group. All participants completed a short face-to-face interview and study questionnaire, which was completed in two periods. For the patient group, there was a pre-procedure period (in the second trimester) and post-procedure period (in the third trimester). For the control group, there was a second trimester and a third trimester.

We divided 45 patients into subgroups by age and number of pregnancy (i.e., ‘first’ alludes to a patient’s first pregnancy). We compared QoL between pre-procedure and post-procedure patients, between each post-operative subgroup, and between patients and healthy controls in the second and third trimesters of pregnancy.

## Statistical analysis

Data are presented as numbers or percentages for categorical variables, while median and interquartile range or mean ± standard deviations were provided for continuous variables. The Mann–Whitney U test or Kruskal–Wallis test was used for continuous variables (pre-procedure data, post-procedure data, subgroup data, and healthy control data). Analysis was performed with SPSS version 22.0 (IBM Corp., Armonk, NY, USA). A *p* < 0.05 was set as statistically significant.

## Results

Among the 45 participants, 26 were aged 20–30 years and 23 were on their first pregnancy. There was no difference between patients and healthy controls, with the exception of cardiac function classification by the New York Heart Association (NYHA) Functional Classification (Table 1). All post-procedure patients had improved functional status to a NYHA classification of Class I. The end-systolic length and width of the right atrium, the right ventricular systolic pressure, and the right ventricular Tei index were also decreased following the procedure. Additionally, the right ventricular ejection fraction was improved following the procedure. (*p* < 0.05) (Table 2).

**Table 2:** Pre-procedure and post-procedure data of patients with atrial septal defect in pregnancy

Characteristics	Preo-procedure data	Posto-procedure data	*P* value
End-systolic length of RA (mm)	49.0 (47.0, 54.0)	41.0 (40.0, 44.5)	< 0.001
End-systolic width of RA (mm)	50.0 (46.0, 52.0)	40.0 (38.0, 45.0)	< 0.001
Right ventricular systolic pressure (mm)	40.0 (37.0, 42.0)	26.0 (23.6, 30.0)	< 0.001
RVEF (%)	55.0 (52.0, 57.0)	59.0 (58.0. 61.0)	< 0.001
RV-Tei	0.47 (0.42, 0.51)	0.35 (0.32, 0.39)	< 0.001
LVEF (%)	60.0 (58.0, 65.5)	63.0 (60.0, 67.0)	0.009

RA: right atrium, RVEF: right ventricular ejection fraction, RV-Tei: right ventricular Tei index, LVEF: left ventricular ejection fraction

Pregnant women with ASD performed better in several measures post-procedure. Table 3 showed the significant differences between scores for pre-procedure and post-procedure data on all scales with the exception of role emotional and mental health (*p* < 0.05). In some domains (physical functioning, bodily pain, general health, vitality, and social functioning), there was a significant difference between the pre-procedure group and the healthy control group in the second trimester of pregnancy (*p* < 0.05) (Table 4). There was no difference between the post-procedure group and healthy pregnant controls in the third trimester (Table 5). In the subgroup analysis, the post-procedure participants in the 31–40 years of age group and the second or third pregnancy group scored better than the patients in the 20–30 years of age group and the first pregnancy group for the social functioning and role emotional dimensions (*p* < 0.05; Tables 6 and 7). In the 20–30 years of age subgroup and the first pregnancy subgroup, the healthy pregnant control group scored better than the post-procedure group for the role emotional dimension. (*p* < 0.05; Tables 8 and 10). In the 31–40 years of age subgroup and the second or third pregnancy subgroup, there was no difference between the healthy pregnant control group and the post-procedure group (Tables 9 and 11).

**Table 3:** Pre-procedure (at 2rd trimester of pregnancy) and post-procedure (at 3rd trimester of pregnancy) SF-36 scores

HRQoL domain	Preo-procedure data	Post-procedure data	*P* value
Physical functioning	51.0 (50.0, 53.0)	72.0 (71.0, 78.0)	< 0.001
Role-physical	45.0 (39.5, 48.0)	32.0 (31.0, 38.0)	< 0.001
Bodily pain	65.0 (60.0, 70.0)	70.0 (65.0, 71.0)	0.012
General health	45.0 (37.0, 47.0)	70.0 (65.5, 72.0)	< 0.001
Vitality	46.0 (42.0, 50.0)	61.0 (57.0, 65.0)	< 0.001
Social functioning	59.5 (55.0, 63.0)	64.5 (63.0, 69.0)	< 0.001
Role-emotional	57.0 (53.0, 65.0)	61.0 (56.0, 67.0)	0.10
Mental health	64.0 (61.0, 67.0)	57.0 (54.0, 67.0)	0.79

**Table 4:** SF-36 scores in pre-procedure patients (2rd trimester of pregnancy) and in healthy pregnant controls (2rd trimester of pregnancy)

HRQoL domain	Patients before procedure (*n* = 45)	Controls (*n* = 45)	*P* value
Physical functioning	51.0 (50.0, 53.0)	72.0 (66.0, 76.0)	< 0.001
Role-physical	41.0 (36.0, 47.0)	45.0 (39.5, 48.0)	0.071
Bodily pain	65.0 (60.0, 70.0)	71.0 (68.0, 75.5)	< 0.001
General health	45.0 (37.0, 47.0)	76.0 (69.5, 79.0)	< 0.001
Vitality	46.0 (42.0, 50.0)	66.0 (63.0, 70.0)	< 0.001
Social functioning	63.0 (59.5, 67.0)	71.0 (67.0, 73.0)	< 0.001
Role-emotional	65.0 (57.0, 70.5)	69.0 (65.0, 71.0)	0.06
Mental health	67.0 (64.0, 72.5)	69.0 (66.0, 71.0)	0.82

**Table 5:** SF-36 scores in post-procedure patients (3rd trimester of pregnancy) and in healthy pregnant controls (3rd trimester of pregnancy)

HRQoL domain	Post-procedure patients (*n* = 45)	Controls (*n* = 45)	*P* value
Physical functioning	67.0 (65.0, 72.0)	68.0 (66.0, 73.0)	0.10
Role-physical	33.0 (31.5, 40.0)	32.0 (31.0, 38.0)	0.22
Bodily pain	68.0 (65.5, 71.0)	70.0 (65.0, 71.0)	0.47
General health	73.0 (68.5, 79.0)	72.0 (70.0, 76.0)	0.43
Vitality	61.0 (59.0, 65.5)	61.0 (57.0, 65.0)	0.67
Social functioning	68.0 (62.5, 71.0)	69.0 (64.5, 72.5)	0.15
Role-emotional	69.0 (64.0, 72.0)	67.0 (61.0, 79.0)	0.80
Mental health	69.0 (64.5, 71.0)	67.0 (57.0, 77.0)	0.79

**Table 6:** SF-36 scores in subgroup after at post-procedue (3rd trimester of pregnancy) in different age groups

HRQoL domain	Age (years) 20–30 (*n* = 26)	Age (years) 31–40 (*n* = 19)	*P* value
Physical functioning	72.0 (70.75, 75.5)	72.0 (71.0, 79.0)	0.42
Role-physical	34.0 (30.75, 38.5)	32.0 (31.0, 38.0)	0.73
Bodily pain	71.0 (62.25, 72.75)	69.0 (65.0, 71.0)	0.25
General health	72.0 (69.0, 75.0)	73.0 (70.0, 78.0)	0.42
Vitality	61.0 (55.0, 65.0)	61.0 (58.0, 67.0)	0.17
Social functioning	61.0 (56.75, 67.0)	79.0 (78.0, 80.0)	< 0.001
Role-emotional	61.0 (55.75, 67.0)	78.0 (73.0, 79.0)	< 0.001
Mental health	69.0 (64.0, 71.0)	72.0 (65.0, 73.0)	0.23

**Table 7:** SF-36 scores in subgroup at post-procedure (3rd trimester of pregnancy) in different number of pregnancies

HRQoL domain	Number of pregnancy First (*n* = 23)	Number of pregnancy Second or third (*n* = 22)	*P* value
Physical functioning	72.0 (71.0, 75.0)	72.0 (71.0, 79.0)	0.36
Role-physical	34.0 (32.0, 40.0)	32.0 (31.75, 36.5)	0.78
Bodily pain	71.0 (63.0, 75.0)	69.0 (65.0, 71.0)	0.095
General health	72.0 (70.0, 75.0)	72.0 (70.0, 78.0)	0.60
Vitality	61.0 (55.0, 65.0)	61.0 (57.0, 67.0)	0.41
Social functioning	61.0 (56.0, 64.0)	79.0 (75.25, 80.0)	< 0.001
Role-emotional	61.0 (55.0, 67.0)	77.0 (72.5, 78.25)	< 0.001
Mental health	69.0 (64.0, 71.0)	70.5 (65.0, 73.0)	0.33

**Table 8:** SF-36 scores in 20–30 years of age subgroup between post-procedure group and healthy pregnant control group

HRQoL domain	Post-procedure group (*n* = 26)	Control group (*n* = 24)	*P* value
Physical functioning	70.0 (68.0, 73.0)	68.0 (65.0, 72.0)	0.12
Role-physical	35.0 (33.0, 38.0)	37.0 (35.0, 40.5)	0.23
Bodily pain	71.0 (63.0, 72.0)	67.0 (65.0, 71.0)	0.14
General health	72.0 (70.0, 75.0)	72.0 (69.5, 79.0)	0.45
Vitality	61.0 (55.0, 65.0)	61.0 (59.0, 65.5)	0.35
Social functioning	69.0 (64.0, 71.0)	70.0 (64.0, 72.0)	0.63
Role-emotional	61.0 (57.0, 64.0)	69.0 (65.0, 74.0)	< 0.001
Mental health	67.0 (61.0, 71.0)	69.0 (64.0, 71.0)	0.51

**Table 9:** SF-36 scores in 31–40 years of age subgroup between post-procedure group and healthy pregnant control group

HRQoL domain	Post-procedure group (*n* = 19)	Control group (*n* = 21)	*P* value
Physical functioning	68.0 (65.0, 73.5)	67.0 (66.0, 71.0)	0.35
Role-physical	36.0 (33.0, 38.0)	38.0 (32.0, 41.0)	0.15
Bodily pain	69.0 (66.0, 70.5)	69.0 (67.0, 74.0)	0.46
General health	69.0 (66.0, 70.5)	73.0 (67.0, 74.0)	0.95
Vitality	61.0 (58.0, 67.0)	59.0 (59.0, 65.0)	0.67
Social functioning	68.0 (65.0, 73.0)	67.0 (62.0, 69.0)	0.61
Role-emotional	70.0 (65.0, 80.0)	69.0 (63.0, 71.0)	0.11
Mental health	70.0 (65.5, 78.5)	69.0 (65.0, 71.0)	0.21

**Table 10:** SF-36 scores in first pregnancy subgroup between post-procedure group and healthy pregnant control group

HRQoL domain	Post-procedure group (*n* = 23)	Control group (*n* = 26)	*P* value
Physical functioning	70.0 (66.0, 75.0)	68.5 (65.0, 72.0)	0.23
Role-physical	34.5 (31.5, 39.0)	37.0 (35.0, 40.0)	0.061
Bodily pain	71.0 (66.5, 73.5)	67.5 (65.0, 71.0)	0.078
General health	72.0 (70.0, 75.0)	72.0 (70.0, 79.0)	0.39
Vitality	61.0 (55.0, 65.0)	61.0 (59.0, 66.0)	0.42
Social functioning	69.0 (64.0, 71.0)	69.5 (63.0, 72.0)	0.89
Role-emotional	61.0 (56.5, 64.0)	69.0 (65.0, 75.0)	< 0.001
Mental health	67.0 (59.5, 70.5)	69.0 (65.0, 71.0)	0.081

**Table 11:** SF-36 scores in second or third pregnancy subgroup between post-procedure group and healthy pregnant control group

HRQoL domain	Post-procedure group (*n* = 22)	Control group (*n* = 19)	*P* value
Physical functioning	72.0 (71.0, 79.0)	70.0 (65.5, 77.5)	0.13
Role-physical	32.0 (32.0, 36.0)	35.0 (32.0, 41.0)	0.082
Bodily pain	69.0 (65.0, 71.0)	69.0 (66.5, 74.0)	0.35
General health	72.0 (70.0, 78.0)	73.0 (67.0, 78.0)	0.99
Vitality	61.0 (57.0, 67.0)	59.0 (58.5, 64.0)	0.81
Social functioning	70.5 (65.0, 73.0)	69.0 (63.5, 72.5)	0.35
Role-emotional	79.0 (76.0, 80.0)	77.0 (69.0, 78.0)	0.45
Mental health	77.0 (73.0, 78.0)	73.0 (68.5, 77.0)	0.21

## Discussion

This study found that QoL in pregnant women with ASD was improved overall following percutaneous closure of ASD with guidance by transthoracic echocardiography.

ASD was a common congenital heart disease for which percutaneous closure was the first-line procedure. However, patients with ASD who were pregnant comprise a special group. In pregnancy, circulating blood volume could increase, which, when combined with a left-to-right shunt can increase the load of the right ventricle and, in cases of large ASD, could lead to pulmonary hypertension and right ventricular dysfunction. Current guidelines recommended cardiological surveillance for all pregnant women with ASD as they were at risk (albeit a small risk) of paradoxical embolism, arrhythmia, and heart failure [8]. Further, percutaneous closure of ASD was an effective and safe procedure for this issue; however, although the time of X-ray fluoroscopy was very short, lasting only a few minutes, and the abdomen was covered with lead clothing, pregnant women were quite often concerned that X-rays will harm the fetus. Thus, guidance by transthoracic echocardiography had been developed for the safe and effective percutaneous closure of ASD [9, 10] as this procedure did not require X-ray fluoroscopy, eliminating radiation concerns.

In this study, we found that QoL in pregnant women with ASD before procedure was lower than that of healthy pregnant controls (in their second trimester). Additionally, QoL was greatly improved following the procedure and was no different from that of healthy pregnant women (in their third trimester). Pregnant women might experience nervousness and depression due to decreased cardiac function or fear of harm to the fetus, thus having a reduced QoL. In our study, participants with ASD underwent successful procedure without complications (arrhythmias, embolisms, or residual shunts). Post-procedure echocardiographic data also showed improvement of right ventricular function. In a previous study, Eroglu et al. [11] reported that the right ventricle was reconstructed 24 h after the closure of ASD, and that the clinical and hemodynamics of patients were improved and maintained during the long-term follow-up period [12, 13]. Additionally, Lazic et al. [14] had reported that the right side of the heart is associated with aerobic capacity. Therefore, owing to the improvement of heart function and the elimination of worry, the overall QoL of pregnant women was expected to improve following surgical closure of the ASD.

In this study, we found that post-procedure pregnant women in the 31–40 years of age and the second or third pregnancy groups had higher scores those in the 20–30 years of age and the first pregnancy groups in the social function and role emotional dimensions. These findings were consistent with previous reports in the literature. For example, García-Blanco et al. [15] reported that social functioning (family functioning, maternal attitudes, and social support) improved with age. McHorney et al. [16] reported that advanced maternal age women was associated with a six or more point increase in the social functioning or role emotional QoL domains. Further, Berryman et al. [17] showed that older pregnant women possessed a greater sense of preparedness and more flexible problem-solving capacities. Thus, it standed to reason that second or third pregnant women would perform better in these aspects due to their experience in pregnancy, suggesting that younger women and those on their first pregnancy should be provided more psychological intervention and social support.

## Limitations

This study was not without limitations. First, the present study was a descriptive study with a limited sample size, which only reflected the status of pregnant women with ASD in one geographical area. Second, this was cross-sectional research and a longitudinal study should be designed in the future to assess QoL of pregnant individuals with ASD throughout pregnancy.

## Conclusion

The post-procedure QoL of pregnant women with ASD was improved overall following percutaneous closure of ASD with guidance by transthoracic echocardiography, which might contribute to pregnancy care and postpartum recovery. In a subgroup analysis, younger pregnant women and women on their first pregnancy performed more poorly in the social functioning and role emotional dimensions, suggesting that these groups require more active psychological and physical support.

## Data Availability

All data generated or analysed during this study are included in this published article.
